# Supplementary material for: How value-sensitive design can empower sustainable consumption
Source: R Soc Open Sci. 2021 Jan 13;8(1):201418. doi: 10.1098/rsos.201418 (PMC7890503; doi:10.1098/rsos.201418)
Supplement: Supplementary Information: How Value-Sensitive Design Can Empower Sustainable Consumption [file rsos201418supp1.pdf]

Supplementary Information:
How Value-Sensitive Design Can Empower Sustainable Consumption

Thomas Asikis[1], Johannes Klinglmayr[2], Dirk Helbing[1] and Evangelos Pournaras[3]

[1]Professorship of Computational Social Science, ETH Zurich, Zurich, Switzerland, E-mail: {asikist,d.helbing}@ethz.ch
[2]Linz Center of Mechatronics GmbH, Linz, Austria, E-mail: johannes.klinglmayr@lcm.at
[3]School of Computing, University of Leeds, Leeds, UK, E-mail: e.pournaras@leeds.ac.uk

Supplementary Figures

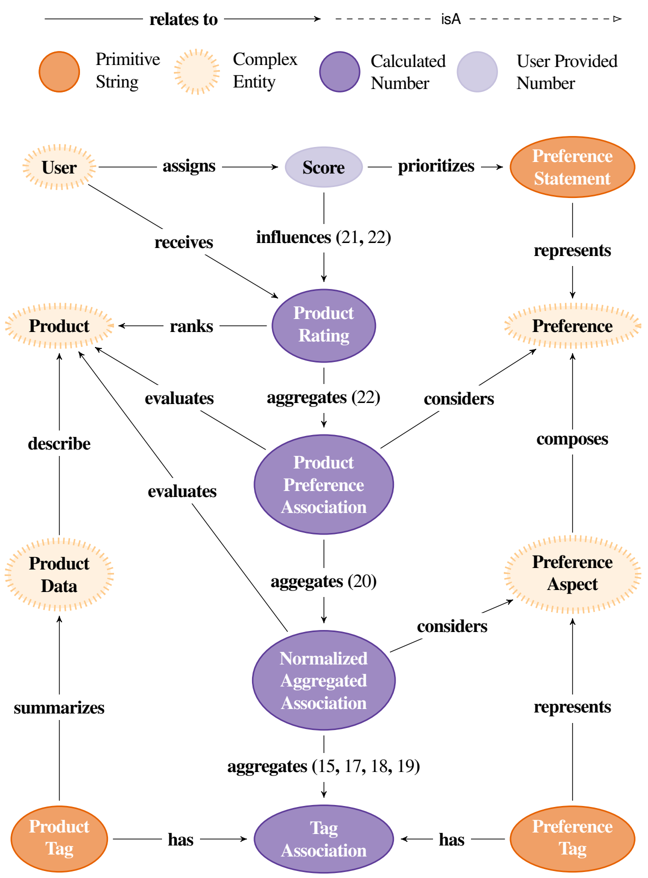

Supplementary Figure S.1: Product rating is calculated by utilizing the ontology. There are ontology relations that contain the relevant equation from the current article in parenthesis.

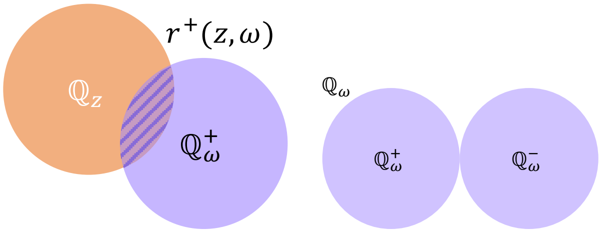

(a) Overlapping product tag and preference tag concepts.

(b) The semantic space that defines a preference tag is the union of the concepts, which support or oppose its existence.

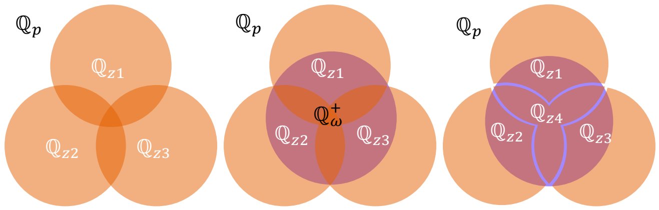

(c) A product concept as a union of its product tag concepts.

(d) Semantic overlap between a product and a preference tag.

(e) Introduction of new product tag according to the reduction design principle.

Supplementary Figure S.2: Venn diagrams that illustrate the operations between sets of preference and product tags primitive concepts.

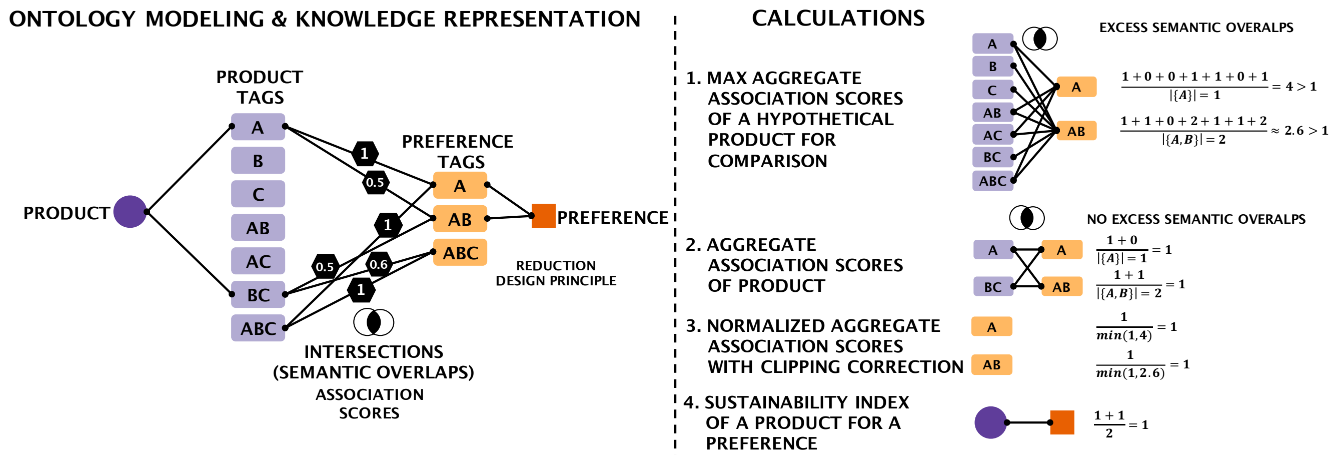

Supplementary Figure S.3: An alternative calculation of sustainability index illustrated in Figure 5. This calculation uses a theoretical normalized aggregate association score that does not respect the reduction design principle of Supplementary Section SM.2.1. *To avoid violation of the bounding properties of Eq.* 19, *the clipping methodology introduced in Supplementary Section* SM.2.5 *is used.*

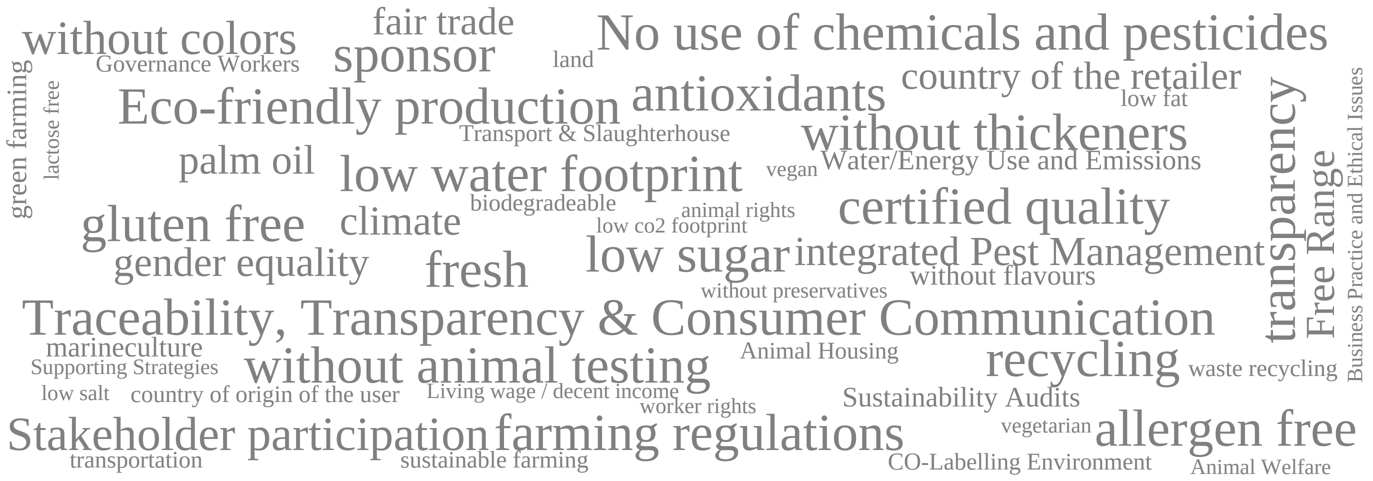

(a) Preference tags.

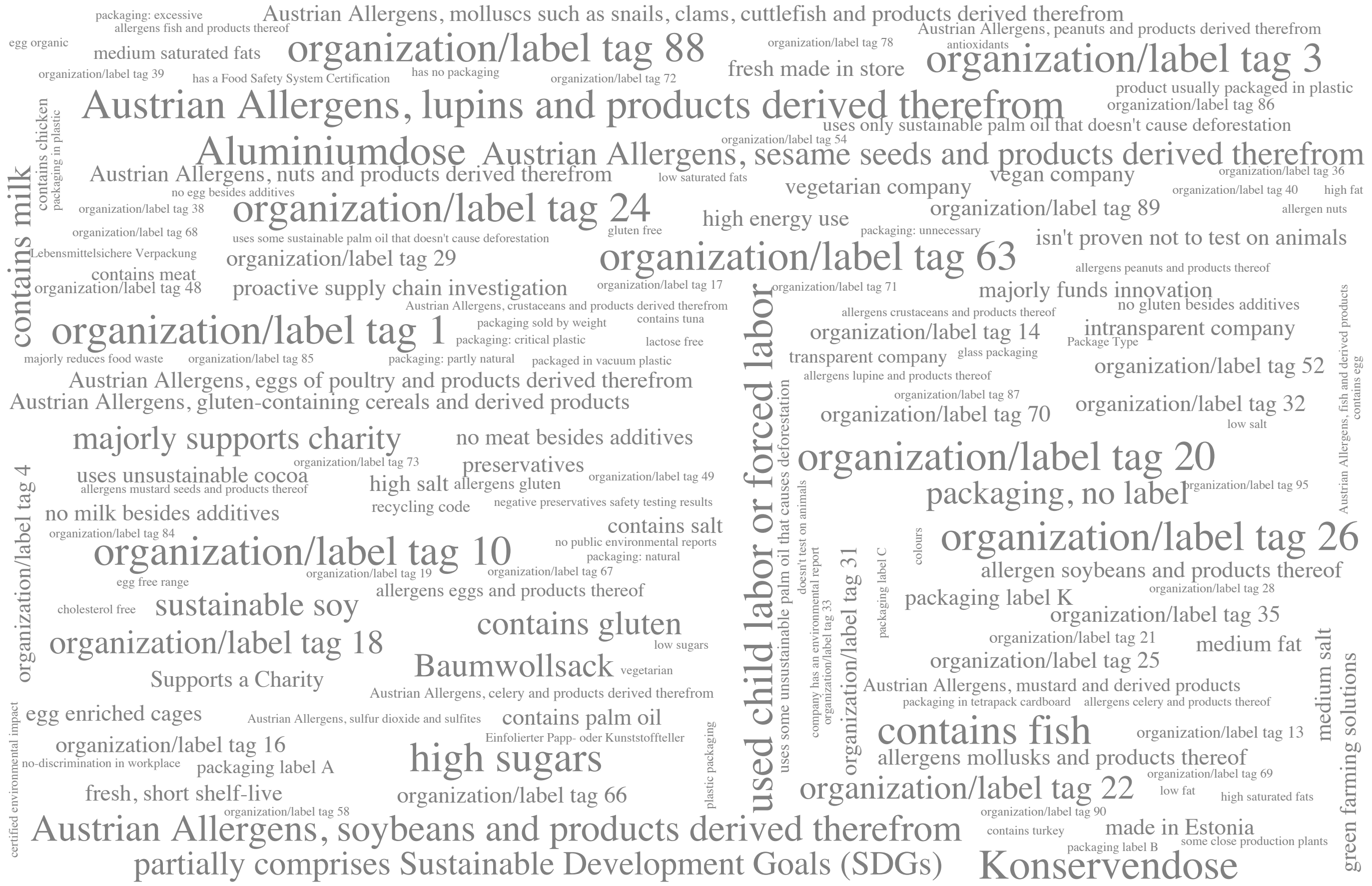

(b) Product tags.

Supplementary Figure S.4: Wordclouds of preference and product tags. The size is proportional to the number of products assigned to each tag.

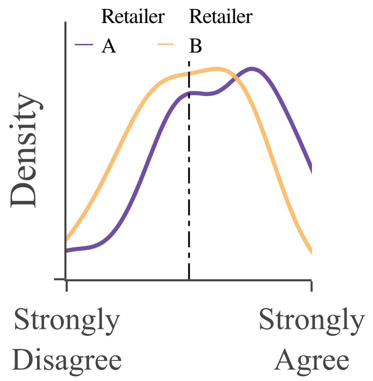

(a) B.I.7: Finding additional product information in the app was easy.

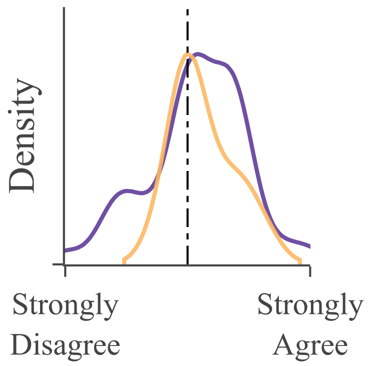

(b) B.I.8: The product info justify the rating of the products.

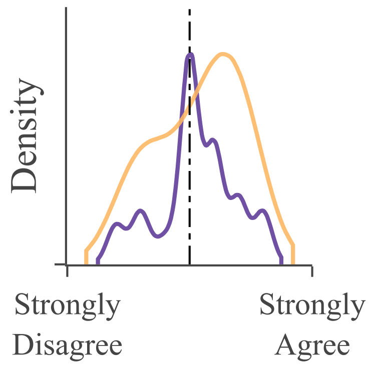

(c) B.II.6: The products with high rating match my preferences.

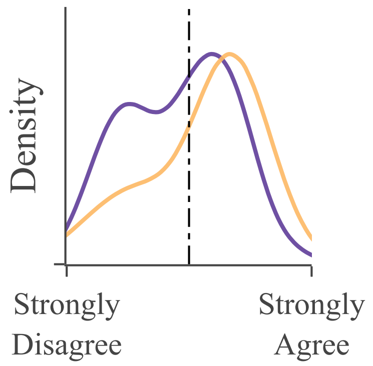

(d) B.II.13: I discovered new products while using the rating functionality.

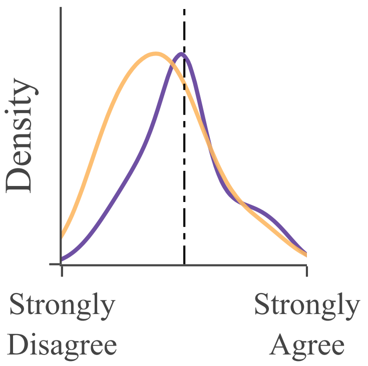

(e) B.II.14: The app does not take into account my preferences when calculating the ratings.

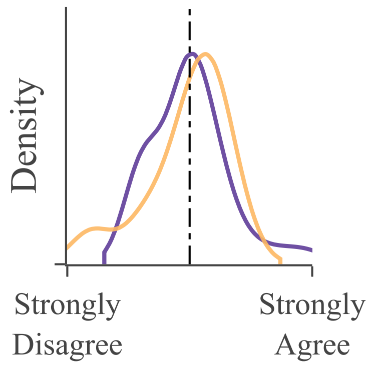

(f) B.III.1: The app could capture my actual preferences.

Supplementary Figure S.5: Questions regarding the application that indicate acceptance from users.

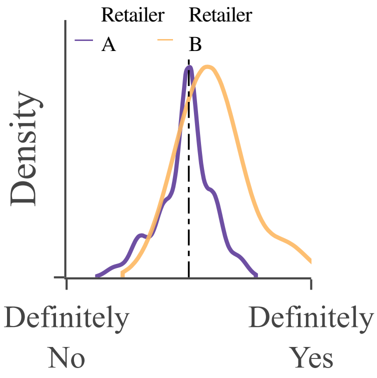

(a) B.IV.3: The products I used to buy before using the app achieve high rating in the app.

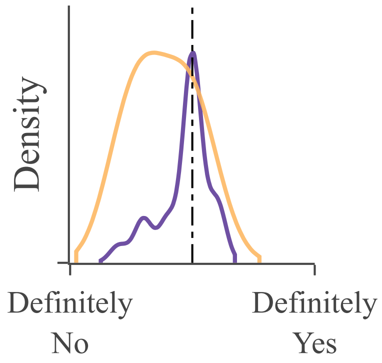

(b) B.IV.4: The products I used to buy before using the app achieve low rating.

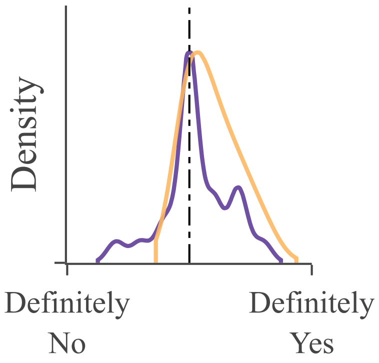

(c) B.IV.5: The products I will buy in the future are the ones with high rating in the app.

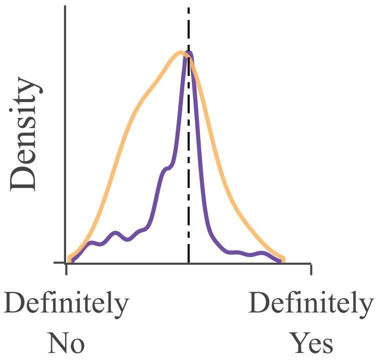

(d) B.IV.6: The products I will buy in the future are the ones with low rating in the app.

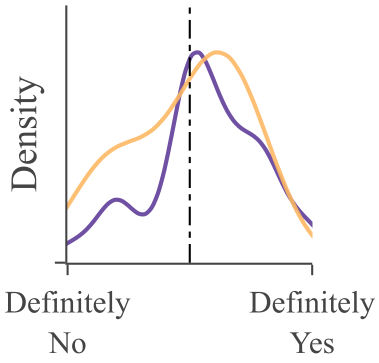

(e) B.IV.9: I am more aware about sustainability aspects after using the app.

Supplementary Figure S.6: Questions regarding sustainability awareness and future purchases.

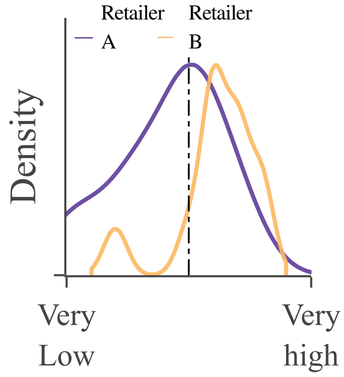

(a) The rating of the app.

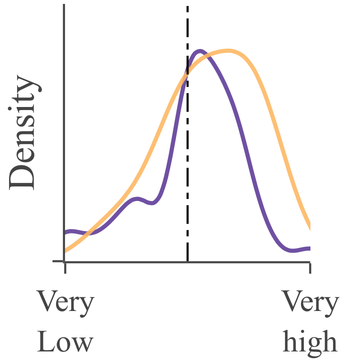

(b) The product information I read in the app.

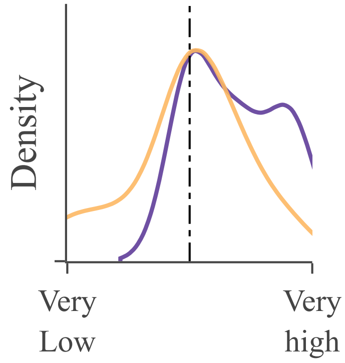

(c) Their price.

Supplementary Figure S.7: The products I finally chose during this study are because of:

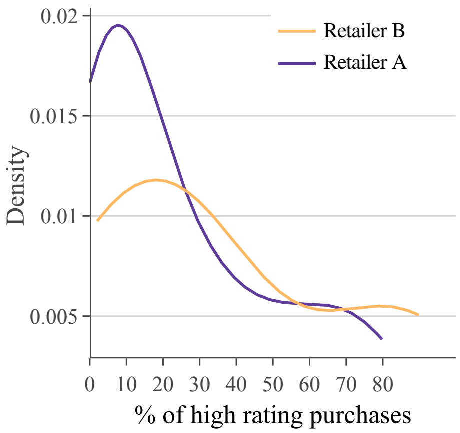

Supplementary Figure S.8: Estimate the % of highly rated products you finally bought:

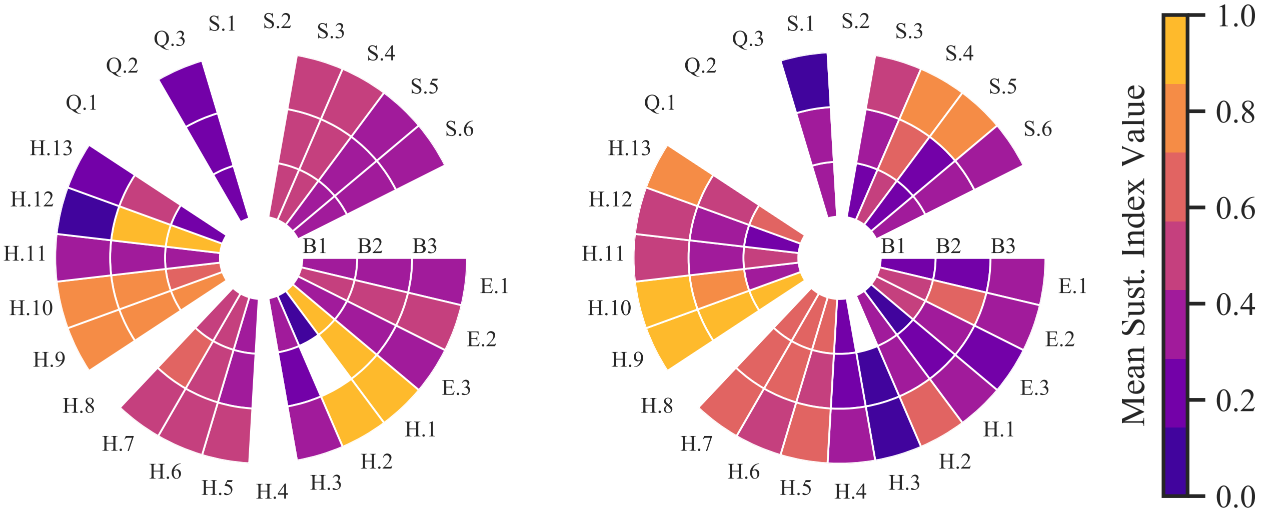

(a) Retailer A. (b) Retailer B.

Supplementary Figure S.9: Evaluation of mean normalized sustainability index (colorscale) per category across the different price bins per category as denoted in Supplementary Table S.11 *for all products of each retailer.*

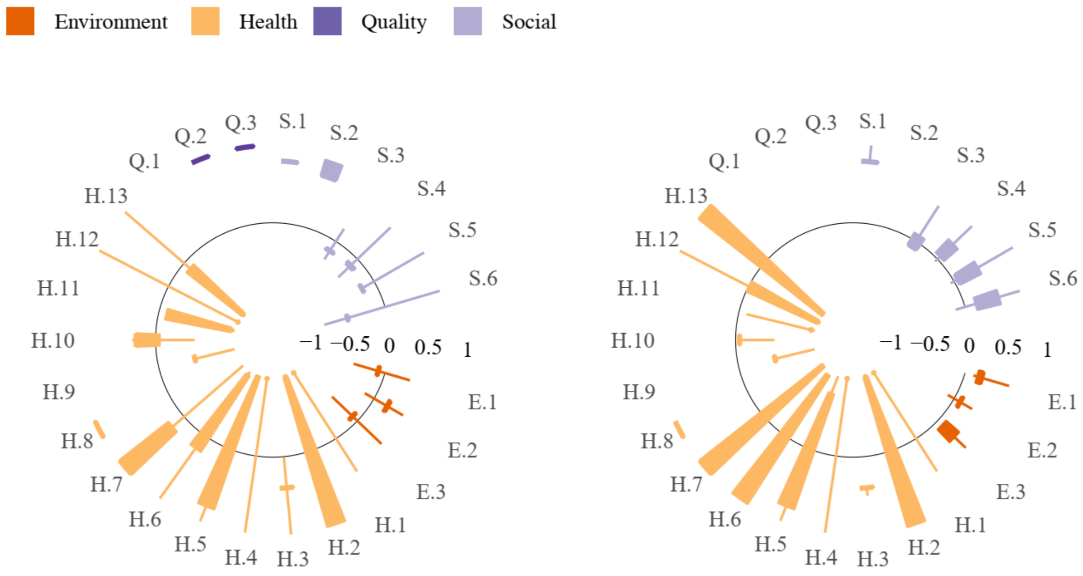

Supplementary Figure S.10: The distribution of sustainability index per preference for all products in both retailers.

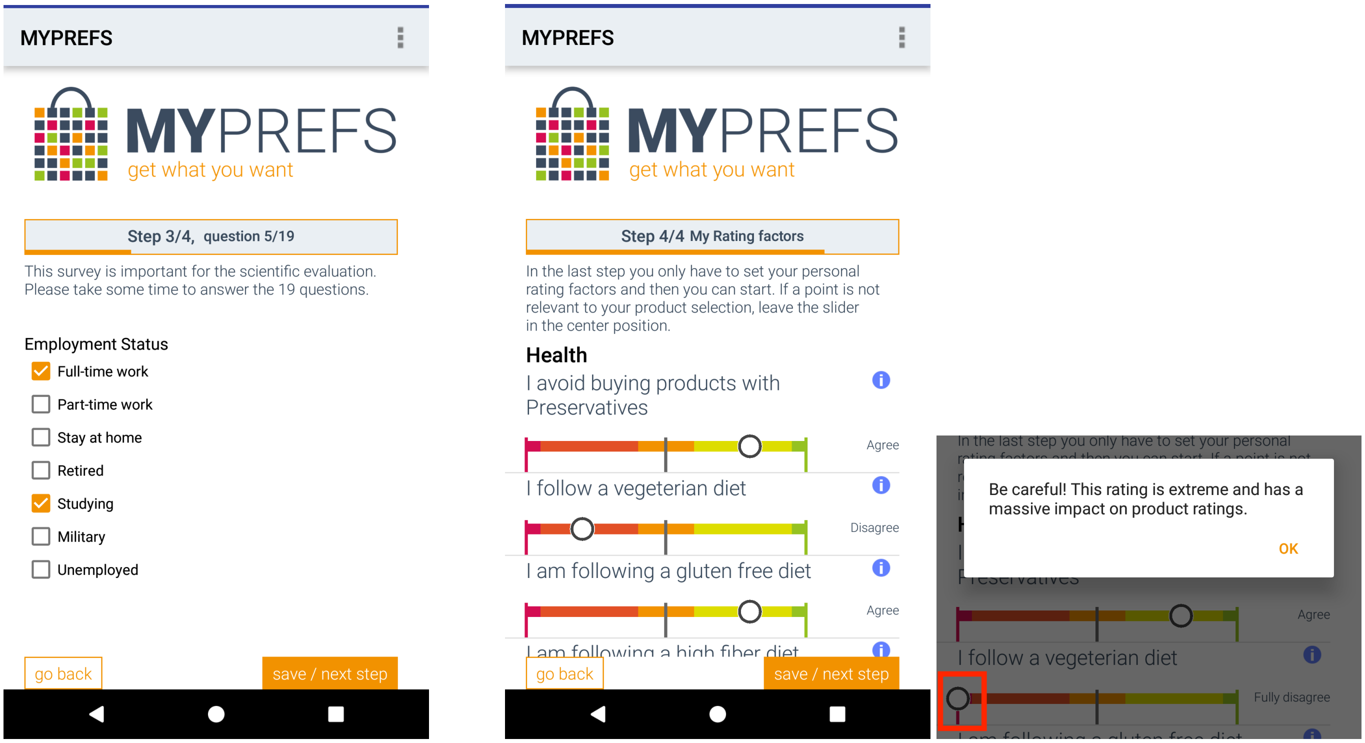

(a) Answering the demographics questions in the entry survey.

(b) Assigning preference scores.

(c) Notification for extreme preference scores, which are also explained to the user in the app tutorial.

Supplementary Figure S.11: Survey and the preference user interfaces of ASSET.

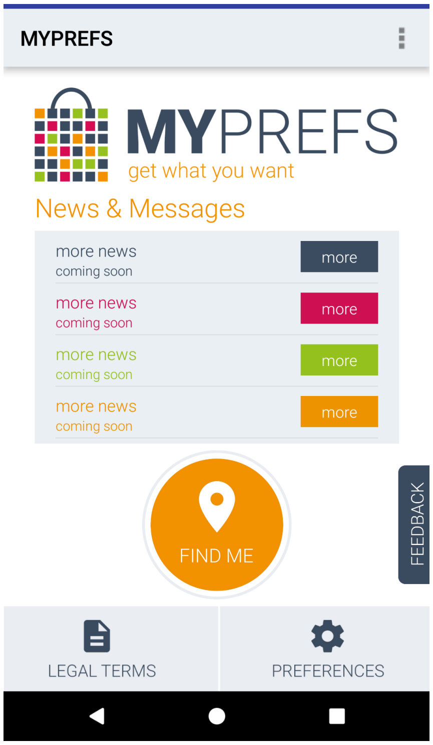

(a) The main screen of the application.

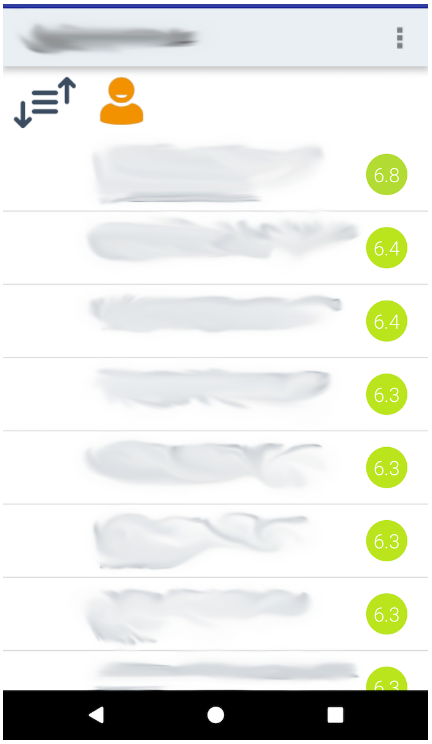

(b) Presenting nearby product categories.

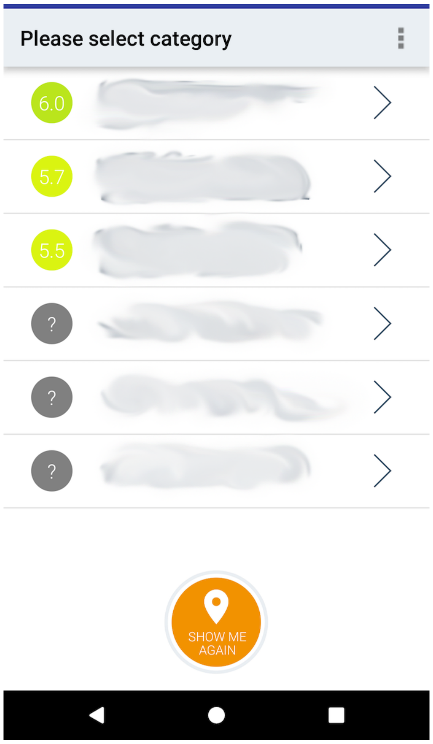

(c) Receiving product ratings.

Supplementary Figure S.12: Using the application.

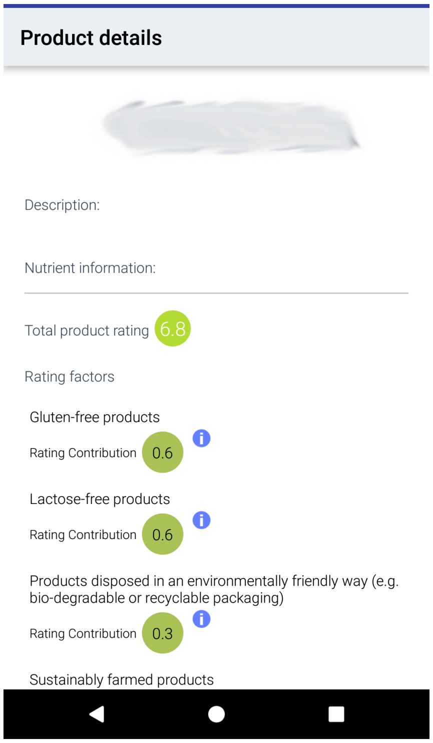

(a) The amount a preference contributes to the product rating.

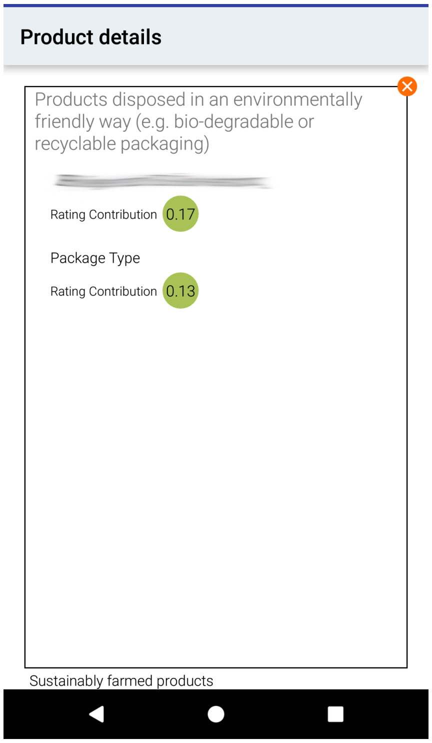

(b) Product tags that contribute a positive amount to the rating.

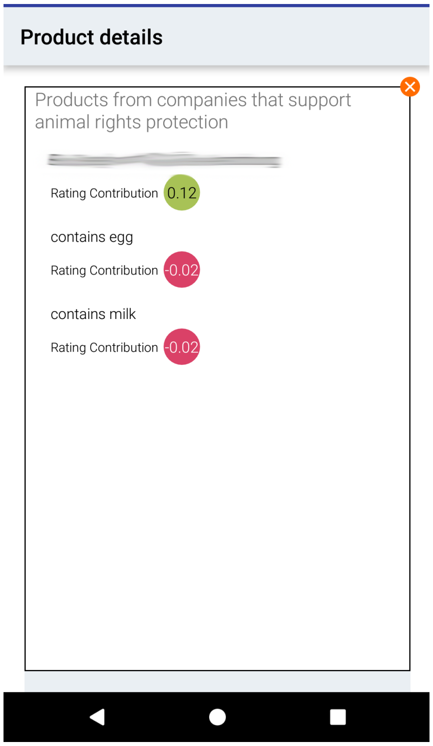

(c) Product tags that contribute a negative amount to the rating.

Supplementary Figure S.13: Preference and product tag contribution to the rating. This feature improves the explainability of the product rating.

Supplementary Tables

Supplementary Table S.1: **Environmental Preferences**. *The preferences setting options provided in value-sensitive smartphone application.*

**Preference**	Description	**ID**
1. Products disposed in an environmentally friendly way (e.g. bio-degradable or recyclable packaging)	Products and product packages that can be recycled efficiently or are biodegradable and do not cause environmental hazard when disposed of.	E.1
2. Products produced and distributed in an environmentally friendly way	A product has a sustainable lifecycle if during manufacturing, distribution, consumption and disposal: it has low CO2 and water footprint, low transportation costs, it can be efficiently recycled and/or biodegradable and produces no waste that is harmful to the environment. Some examples include toxic waste, high CO2 emissions during production, usage of palm oil during production that leads to deforestation etc.	E.2
3. Sustainably farmed products	Sustainable farming is achieved by a company by applying farming methods such as Integrated Pest Management, No-till agriculture, biodynamic and permaculture. For sea products, aquaculture is preferred instead of free fishing. The countries where the company produces its products should have clear and fair regulations regarding farming and land-management.	E.3

Supplementary Table S.2: **Quality Preferences**. *The preferences setting options provided in value-sensitive smartphone application.*

**Preference**	Description	**ID**
1. Award winning or high quality certified products.	There are several officially recognized organizations that offer awards and/or certifications to companies that produce high-quality products.	Q.1
2. Fresh products	Fresh products are directly brought from production to the shelves of the supermarket. Fresh products usually tend to be richer in nutrients. Also it is less likely for fresh products to cause health problems to the digestive system.	Q.2
3. Locally originated and domestic products.	I prefer products that the country of origin is the same as the one I am living in.	Q.3

Supplementary Table S.3: **Social Preferences**. *The preferences setting options provided in value-sensitive smartphone application.*

**Preference**	Description	**ID**
1. Products evaluated with auditing processes that rely on sustainability criteria.	All the processes that enhance and enable sustainability should be checked and validated from 3rd parties. Auditing processes based on sustainability criteria support companies in adopting more sustainable processes and also increase their transparency.	S.1
2. Products from companies that actively contribute to public and social good	I prefer supporting companies, that sponsor charities, scholarships, R&D, social activities	S.2
3. Products from companies that support animal rights protection	Brands that respect and support animal rights should avoid factory farming, animal testing and any kind of animal abuse/mistreatment during the production of their products.	S.3
4. Products from companies that support fairness and equality in the workplace	Brands that treat their workers equally and respect their rights. Such brands should take action against any discrimination between their employees and promote gender and race equality.	S.4
5. Products from companies with transparent activities	I prefer buying products from companies that are open to sharing the impact and nature of their operation.	S.5
6. Products from fair trade label companies	Fair trade focuses on human rights in general. Companies should respect human rights and trade with producers in developing and developed countries in a fair manner, offering fair prices for raw materials and not abusing local laws for fair resource sharing, like public water sources, fair land management etc.	S.6

Supplementary Table S.4: **Health Preferences**. *The preferences setting options provided in value-sensitive smartphone application.*

**Preference**	Description	**ID**
1. Allergen-free products	Food allergens are typically naturally-occurring proteins in foods or derivatives of them that cause abnormal immune responses. Eight ingredients cause about 90% of food allergy reactions: Milk (mostly regarding children), Eggs, Peanuts, Tree nuts, like walnuts, almonds, pine nuts, brazil nuts, and pecans, Soy, Wheat and other grains with gluten, including barley, rye, and oats, Fish (mostly in adults), Shellfish (mostly in adults)	H.1
2. Gluten-free products	A gluten-free diet is a diet that strictly excludes gluten, a mixture of proteins found in wheat and related grains, including barley, rye, oat, and all their species and hybrids (such as spelt, kamut, and triticale). The inclusion of oats in a gluten-free diet remains controversial, and may depend on the oat cultivation and the frequent cross-contamination with other gluten-containing cereals.	H.2
3. High-protein products	Protein is one of the three macronutrients, along with carbs and fat. It is beneficial for building muscle. Protein serves a number of important functions in your body. It is made up of individual amino-acids, including many that your body cannot create on its own. Protein is the main component of your muscles, bones, skin and hair. These tissues are continuously repaired and replaced with new protein.	H.3
4. Lactose-free products	A lactose free diet means eating foods that have no lactose. Lactose is a sugar that is a normal part of dairy products. Some people do not break down lactose well. They may not have enough lactase, the enzyme that breaks lactose down in the body. Or, their body may create lactase variants that do not work properly.	H.4
5. Low fat products	Fats are a type of nutrient that you get from your diet. It's a major source of energy. It helps you absorb some vitamins and minerals. Fat is needed to build cell membranes, the vital exterior of each cell, and the sheaths surrounding nerves. It is essential for blood clotting, muscle movement, and inflammation. It is essential to eat some fats, though it is also harmful to eat too many. For long-term health, some fats are better than others. Good fats include monounsaturated and polyunsaturated fats. Bad ones include industrial-made trans fats. Saturated fats fall somewhere in the middle. Saturated fats raise your LDL (bad) cholesterol level. High LDL cholesterol puts you at risk for heart attack, stroke, and other major health problems. Trans fats can raise LDL cholesterol levels in your blood. They can also lower a person's HDL (good) cholesterol levels.	H.5
6. Low salt products	There is also some evidence that too much salt can damage the heart, aorta, and kidneys without increasing blood pressure, and that it may be harmful for bones, too.	H.6
7. Low sugar products	Added sugar is known to cause heart diseases. Sugar delivers "empty calories" — calories unaccompanied by fiber, vitamins, minerals, and other nutrients. Too much added sugar can replace healthier foods from a person's diet.	H.7
8. Products rich in antioxidants	Antioxidants come up frequently in discussions about good health and preventing diseases. Their nature is to prohibit (and in some cases even prevent), the oxidation of other molecules in the body. Oxidation is a chemical reaction that can produce free radicals, leading to chain reactions that may damage cells. The term "antioxidant" is mainly used for two different groups of substances: industrial chemicals which are added to products to prevent oxidation, and natural chemicals found in foods and body tissue which are said to have beneficial health effects. It is often debated whether they actually prevent diseases, which antioxidant(s) are needed from the diet and in what amounts beyond typical dietary intake.	H.8
9. Products without artificial colours or flavor enhancers.	Artificial colors and flavoring enhancers are used to produce coloring effects and improve food taste. In general these substances are exhaustively tested in labs before they are used in food production. Still, it is not fully determined whether they cause health problems in the long term.	H.9
10. Products without preservatives	A preservative is a substance or a chemical that is added to products such as food, beverages, pharmaceutical drugs, paints, biological samples, cosmetics, wood, and many other products to prevent decomposition by microbial growth or by undesirable chemical changes. Preservatives are used to prolong the shelf-life of the product but may cause health problems in the long term.	H.10
11. Products without thickeners, stabilizers or emulsifiers	Emulsifiers allow water and oils to remain mixed together in an emulsion, as in mayonnaise, ice cream, and homogenised milk. Stabilizers, thickeners and gelling agents, like agar or pectin (used in jam for example) give foods a firmer texture. While they are not true emulsifiers, they help to stabilize emulsions. These additives may cause health problems in the long term.	H.11
12. Vegan products	Vegans choose not to consume dairy, eggs or any other products of animal origin, in addition to not eating meat like the vegetarians. Veganism was originally defined as "the principle of emancipation of animals from exploitation by man."	H.12
13. Vegetarian products	The Vegetarian Society defines a vegetarian as follows: "A vegetarian is someone who lives on a diet of grains, pulses, legumes, nuts, seeds, vegetables, fruits, fungi, algae, yeast and/or some other non-animal-based foods (e.g. salt) with, or without, dairy products, honey and/or eggs. A vegetarian does not eat foods that consist of, or have been produced with the aid of products consisting of or created from, any part of the body of a living or dead animal. This includes meat, poultry, fish, shellfish*, insects, by-products of slaughter** or any food made with processing aids created from these."	H.13

Supplementary Table S.5: An example of association score values and their corresponding meaning. RDA refering to "Recommended Daily Allowance" [1]. LDL refering to "Low-density lipoprotein".

association score	product tag $\boldsymbol{z}$	preference tag $\boldsymbol{\omega}$	**Meaning**
$r(z,\omega)=1$	vegetable	vegetarian diet	p fully supports c via z
$r(z,\omega)\in(0,1)$	10% RDA Vitamin C	healthy diet	p partially supports c via z
$r(z,\omega)=0$	contains sugar	animal rights	z is irrelevant to ω
$r(z,\omega)\in(-1,0)$	contains LDL	healthy diet	ω partially opposes z
$r(z,\omega)=-1$	contains eggs	vegan diet	ω fully opposes z

*Supplementary Table S.6: **Alternative approaches.** Overview of online sources that provide sustainability ratings. Most of these approaches limit their scope to evaluation of brands rather than a broad spectrum of products. They are online web approaches with limited integration to shopping processes in retailer shops. Explainability is not explicitly provided and data may only be collected in a centralized fashion. They do not always capture a broad spectrum of sustainability goals. No rigorous evaluation with field studies has been shown how they impact sustainable consumption.*

Organization	Sector Scope	Target	Computation	Main Focus	Rating Type
rankabrand [2]	General	Brand	Crowdsourced	Environment, Social and Health	Ordinal
GoodGuide® [3]	Personal Care, Household Supplies, Babies and Kids products	Product	Ingredient analysis based on official regulations	Environment and Health	Continuous
Shop Ethical! [4]	General	Brand	Screening and reports for praises and/or criticism towards companies based on expert weighting	Social and Environmental	Ordinal
THE GOOD SHOPPING GUIDE [5]	General	Companies, Brands	Qualitative and quantitative evaluation based on expert criteria.	Environment, Animals, People, Ethical and Other Factors	Ordinal
The Green Stars Project [6]	General	Companies, Brands, Services, Products etc.	User based reviews and rating assignment	Social and Environmental	Ordinal
WikiRate [7]	General	Company	Allows for implementation of any rating based on the data the website offers	Environmental, Social and Governmental Concerns	Any
Behind the Brands [8]	Food	Brand	Expert based	Social, Environmental and Ethical	Continuous, ordinal

*Supplementary Table S.7: **Data Sources**. The data sources used for the construction of the used ontology. Websites accessed on January 2020.*

Source Type	Institute	Website
Retailer Database	Coop Estonia	coop.ee
	Winkler Markt	www.winklermarkt.at
Online Datastores	GS1	gs1.org
	ecoinvent	ecoinvent.org
Experts	AINIA	ainia.es
	BIA	bia.ee
	ETH	ethz.ch
	Ethical Consumer Workshop 2017	ethicalconsumer.org
	LCM	lcm.at
	VKI	vki.at
Crowdsourcing	Wikipedia	wikipedia.org
	Social Impact Data Hack	sidh2017.ut.ee

Supplementary Table S.8: Approximate confidence intervals and error margins calculated based on available sample size. The population size for the calculations is estimated as the average number of loyalty card visitors per month .

Retailer	Survey	# Users
Retailer A	Entry	323
	Exit	44
	Preferences	315
Winkler Markt	Entry	69
	Exit	12
	Preferences	66

*Supplementary Table S.9: **Causal Impact Analysis**. Best kNN matches for treatment group selected on the DTW euclidean distance on high mean rating products weekly expenditure. The control group is selected by using covariates for matching between users. Since different values of k result in different control group per combination of criteria the best value of k is selected via a dynamic time warping of weekly expenditure before treatment. The warping window size is 1, meaning that the algorithm searches 1 week before or after to match the expenditure of the current weekly expenditure between control and treatment.*

Retailer	Monthly Total Expenditure	Distribution of Monthly Expenditure per Category	Monthly Representations	Distance	k
retailer A	-	-	✓	0.030852	5
	-	✓	-	0.037871	5
	-	✓	✓	0.032995	2
	✓	-	-	0.032736	4
	✓	-	✓	0.033386	5
	✓	✓	-	0.033722	2
	✓	✓	✓	0.035229	4
retailer B	-	✓	-	0.056523	1
	-	✓	✓	0.061028	2
	✓	-	-	0.066083	2
	✓	-	✓	0.055792	2
	✓	✓	-	0.061923	1
	✓	✓	✓	0.059597	2

Supplementary Table S.10: The summary of the causal impact analysis for Retailer A and Retailer A users. The reported decimal values in the table are rounded.

**Retailer**		**Average**			**Cumulative**			p
		value	s.d.	95% c.i.	value	s.d.	95% c.i.	
A	Actual	0.8	-	-	14.7	-	-	≤ 0.001
	Prediction	0.6	0.0	[0.5, 0.6]	10.8	0.3	[10.1, 11.4]	
	Absolute Effect	0.2	0.0	[0.2, 0.2]	4.0	0.3	[3.3, 4.6]	
	Relative Effect	36.7%	3.1%	[30.7%, 42.8%]	-	-	-	
B	Actual	0.6	-	-	10.1	-	-	≤ 0.001
	Prediction	0.4	0.0	[0.3, 0.5]	7.1	0.8	[5.4, 8.7]	
	Absolute Effect	0.2	0.0	[0.1, 0.3]	2.9	0.8	[1.4, 4.7]	
	Relative Effect	41.0%	11.8%	[19.2%, 65.4%]	-	-	-	

Supplementary Table S.11: The price bins used for classifying a product according to its price per unit in Supplementary Figure S.9*. Each product price is compared against prices of other products in the same category. Package specific quantities are not taken into account.*

Label	Normalized Price Range	Description
B1	(0,0.33)	The 33% of lowest product prices in category
B2	(0.33,0.66)	Between 33% and 66% product prices
B3	(0.66,1)	The 66% and 100% of highest product prices in category

Supplementary Table S.12: (Max-Min) Normalized price per category and normalized sustainability index for retailer A. Relevant to Supplementary Figure S.9a*.*

Preference	Correlation	p-value
E.1	0.03	0.21
E.2	-0.08	0.00
$^{+}$E.3	0.10	0.00
H.1	0.09	0.70
H.2	1.00	-
H.3	-0.05	0.47
H.4	-	-
H.5	-0.02	0.21
H.6	-0.03	0.17
H.7	-0.04	0.12
H.8	-	-
$^{-}$H.9	-0.10	0.01
H.10	0.14	0.23
H.11	0.03	0.74
$^{-}$H.12	-0.88	0.02
H.13	0.13	0.11
Q.1	-	-
Q.2	-	-
$^{+}$Q.3	0.12	0.01
S.1	-	-
S.2	-	-
S.3	0.00	0.81
S.4	-0.05	0.00
S.5	0.02	0.21
S.6	0.07	0.00

Supplementary Table S.13: (Max-Min) Normalized price per category and normalized sustainability index for retailer B. Relevant to Supplementary Figure S.9b.

Preference	Correlation	p-value
E.1	-0.03	0.03
$^{-}$E.2	-0.17	0.00
$^{-}$E.3	-0.34	0.00
$^{+}$H.1	0.23	0.00
$^{+}$H.2	0.22	0.00
H.3	0.02	0.92
H.4	0.09	0.00
H.5	-0.09	0.00
$^{+}$H.6	0.12	0.00
H.7	-0.01	0.59
H.8	-	-
H.9	-0.03	0.15
$^{+}$H.10	0.40	0.02
H.11	0.01	0.78
$^{+}$H.12	0.17	0.00
$^{+}$H.13	0.20	0.00
Q.1	-	-
Q.2	-	-
Q.3	-	-
$^{-}$S.1	-0.30	0.00
S.2	-	-
$^{+}$S.3	0.29	0.00
$^{+}$S.4	0.22	0.00
$^{+}$S.5	0.35	0.00
S.6	-0.08	0.05

SUPPLEMENTARY METHODS

SM.1 Ontology

In the used product ontology, product characteristics are summarized in the form of words or phrases, the **product tags**. Each product tag z can be assigned to one or more products and it summarizes concepts and characteristics of a product p. Every product p can be assigned to multiple product tags. The set of all product tags denotes the semantic space of product characteristics. Product tags are generated based on the data available from the data sources. For example $p=$"cabbage" is associated with the product tag $z=$"vegetable".

The consumer preferences (see Table S.1-S.4) are also represented in the consumer preference ontology. Several challenges arise when defining an universal golden standard regarding a sustainable consumption behavior. Therefore, a personalized view on sustainable consumption is evaluated per consumer. Each preference is presented to the consumer in form of a statement c. Each statement is accompanied by a description that explains the sustainability concepts composing this preference, e.g. $c=$"I prefer vegan products.". A consumer u then assigns numerical values to each preference statement c, to express support, opposition or neutrality towards the preference statement. This numerical value is referred to as preference score $s_{u,c}$. The preference score is bound in the range of $[0,2\cdot\overline{s}]$, where $\overline{s}$ is the mean value of the range. The minimum value of the score implies that a consumer fully opposes a sustainability preference. The maximum value implies fully supports a sustainability preferences. The mean value of the scale implies no consumer preference regarding a sustainability criterion. An assigned preference score is stored in the device of a consumer u, and represents personalization. The rating system calculates the product rating $\varrho(p,u)$ for a given product p and a consumer u based on the preference scores. The introduced method extends the semantic differential methodology for evaluating associations of product characteristics and sustainability preferences [9], [10].

Preference statements usually express *abstract* complex sustainability aspects. These aspects are decomposed into simpler ones creating a hierarchical ontology. A word or phrase, referred to as preference tag ω, represents an aspect of a preference statement. For example, $c=$ "I prefer products that can be disposed in a sustainable manner." is composed by two sustainability preference tags: $\omega_1=$"biodegradability", which is the ability of the product to dissolve within an acceptable time and without harming the environment [11], and $\omega_2=$"recycling capability", which denotes whether a product can be recycled in an efficient and environmentally friendly way [12]. In other words, a preference tag $\omega\in\Omega_c$, which belongs to a preference tag set Ω_c of a preference c, compresses and represents information regarding the concepts that compose the preference statement c.

The main challenge of designing an ontology for a sustainable consumption ontology is to define quantifiable associations between the semantic spaces of product tags and preference tags. To enable numerical calculation for product ratings we use the ontological design as sketched in *Supplementary Figure* S.1. Several ontological connections are introduced to calculate the aggregate support or opposition of a product to a consumer's preferences.

The ontology of sustainability concepts has hierarchical structures of concepts [13], where one concept is composed of several other concepts $q\in\mathbb{Q}$. If it is not feasible to further decompose a concept, then such a concept is referred to as a *primitive concept*[1]. A **semantic association framework** introduces a logic for quantifiable semantic associations between a product tag z and a preference tag ω. Examples of such associations can be found on *Supplementary Table* S.5. The associations between tags are quantified in a shared semantic space $\mathbb{Q}$, which contains all concepts relevant to products and sustainability preferences. The space is defined with the following two assumptions:

1) The individual elements of this space are *primitive concepts* that cannot be decomposed into other concepts within the defined sustainability scope.
2) Complex concepts are represented as sets that contain all the *primitive concepts* that compose them. For instance, the preference tag $\omega=$ "vegan" may be decomposed to the set $\mathbb{Q}_\omega=\{$ "no animals involved in production", "no animal products involved in production", ...$\}$ where $\mathbb{Q}_\omega\subseteq\mathbb{Q}$.

Suppose a product tag expresses one or more *primitive concepts*. The set of those concepts is defined as $\mathbb{Q}_z\subseteq\mathbb{Q}$. Following the same logic, let a preference that its existence is denoted by the union of *primitive concepts* such as $\mathbb{Q}_\omega^+$. An association between the product and the preference tag is defined as the overlap between the *primitive concepts* that each tag represents $\mathbb{Q}_z\cap\mathbb{Q}_\omega^+$ as shown in *Supplementary Figure* S.2a. This overlap is maximized when all *primitive concepts* that compose the preference tag also compose the product tag. The following positive association score is defined:

$$r^+(z,\omega)=\frac{|\mathbb{Q}_z\cap\mathbb{Q}_\omega^+|}{|\mathbb{Q}_\omega^+|} \tag{1}$$

The score is bounded, since:

$$\mathbb{Q}_z\cap\mathbb{Q}_\omega^+\subseteq\mathbb{Q}_\omega^+\Leftrightarrow r^+(z,\omega)\in[0,1] \tag{2}$$

For example, let a product tag and the relevant *primitive concepts*: $z=$ "vegetable" with $\mathbb{Q}_z=\{$ "minimal CO_2 footprint", "plant part", "not animal product"$\}$. Suppose the following two preference tags, which are decomposed to *primitive concepts*, $\omega_1=$ "vegetarian diet" with $\mathbb{Q}_{\omega1}=\{$ "plant part", "not animal product"$\}$ and $\omega_2=$ "sustainable production" $\mathbb{Q}_{\omega2}=\{$ "minimal CO_2 footprint", "minimal water

[1] The definition of primitive concepts serves more as a theoretical tool, with which the scope of the sustainability can be determined.

footprint", "no toxic waste"}. The "vegetable" product tag is composed of concepts which are enough to guarantee a "vegetarian diet". Yet, the "vegetable" product tag is composed of some but not all of the concepts that guarantee a sustainable production.

A product tag may oppose the existence of a preference tag concept. For example the concept of "animal product" in food, fully opposes a "vegan diet" preference. When a product contains animal products, then it is definitely not vegan. Partial opposition is also possible, as for example the concept "contains lactose" indicates that a product probably contains animal products, as lactose is a protein mainly found in milk [14]. Therefore, the need to define opposite associations arises. The negative *primitive concepts* of a preference tag are defined as a set $\mathbb{Q}_\omega^-$. For that, an association is defined when the concept in a product tag is mutually exclusive with concepts defining a preference tag. In this case a negative association score evaluates the overlap of concepts belonging to the set of simple concepts of the product tag $\mathbb{Q}_z \cap \mathbb{Q}_\omega^-$. The negative association score is defined as:

$$r^-(z,\omega) = \frac{|\mathbb{Q}_z \cap \mathbb{Q}_\omega^-|}{|\mathbb{Q}_\omega^-|} \tag{3}$$

The negative score is also bounded, since:

$$\mathbb{Q}_z \cap \mathbb{Q}_\omega^- \subseteq \mathbb{Q}_\omega^- \Leftrightarrow r^-(z,\omega) \in [0,1] \tag{4}$$

The preference tag is defined by the union of its negative and positive concepts $\mathbb{Q}_\omega = \mathbb{Q}_\omega^+ \cup \mathbb{Q}_\omega^-$ as shown in *Supplementary Figure* S.2b. E.g. the preference tag $\omega=$ "vegan" can be decomposed to the supporting *primitive concepts* $\mathbb{Q}_\omega^+=$ "plant part".

Product tags that contain *primitive concepts* that both oppose and support a preference tag are also possible. In such case, the sum of positive and negative scores is calculated. For this calculation, it is assumed that positive simple concepts cancel out negative concepts and vice versa. The association score between a product tag and a preference tag is defined as:

$$r(z,\omega) = r^+(z,\omega) - r^-(z,\omega) \overset{2,4,5}{\Leftrightarrow} r(z,\omega) \in [-1,1] \tag{5}$$

This is denoted as the **individual bounding property** of the score and applies to all association scores between any product and preference tags.

A product is often related to a set of product tags $\mathbb{Z}_p$, therefore it can be represented by the union of all *primitive concepts* of these tags:

$$\mathbb{Q}_p = \bigcup_{z \in \mathbb{Z}_p} \mathbb{Q}_z \tag{6}$$

An example of how a product p is decomposed to the *primitive concepts* of the product tags it consists of is found in *Supplementary Figure* S.2c.

In practice, the calculation of the association score is performed by knowledge systems that rely on (i) expert knowledge, (ii) crowdsourcing and (iii) machine learning. As illustrated on line 4 in *Supplementary Table* S.5, the products that contain high quantities of LDL, oppose the preference tag "healthy diet", e.g. the health of the cardiovascular system [15]. This is quantified via a negative association score value in the range $(-1,0)$. The negative threshold value is assigned to the "contains eggs" product tag, which fully opposes the "vegan" preference tag, as shown on row 5 of *Supplementary Table* S.5.

Associations between products p and preference tags are calculated by an aggregated association score $\eta(p,\omega)$. According to *Eq.* 6, the intersection between all *primitive concepts* related to a product and a preference tag is used for such calculation:

$$\eta(p,\omega) = \frac{|\mathbb{Q}_p \cap \mathbb{Q}_\omega^+|}{|\mathbb{Q}_\omega^+|} - \frac{|\mathbb{Q}_p \cap \mathbb{Q}_\omega^-|}{|\mathbb{Q}_\omega^-|} \overset{Eq.\ 6}{\Leftrightarrow} \tag{7}$$

Note that, it is challenging to assign *primitive concepts* directly to products. A fixed sustainability scope supports the identification of overlaps and the existence of primitive concepts. The definition of primitive concepts is challenging when the ontology is under construction and the sustainability scope is not fixed. Conceptual overlaps are common in real world scenarios where product tags are usually derived from labels or certifications, which are related to several *primitive concepts*, e.g.the fair trade label. Intersection between a product tag and a preference tag $r(z,\omega)$ quantifies their shared semantic space, consisting of at least one primitive concept. The individual intersections between product tags and preference tags $r(z,\omega)$ can be used for the approximation of the aggregated association $\eta(p,\omega)$. The number of available product tags is considerably lower than the number of products worldwide. When the product tags share *primitive concepts*, it may prove challenging to calculate this intersection, especially when the *primitive concepts* are not identified. Shared product tag *primitive concepts* introduce overlaps between intersections of different product tags and a preference tag. Such overlaps introduce an error ϵ in the approximation of the aggregate associations $\eta(p,\omega)$. Reducing the overlaps between product tags of the same product, minimizes this error, as shown in *Lemma* 1.

Lemma 1. *The aggregated association $\eta(p,\omega)$ between a product p and a preference tag ω is approximated with error ϵ by the sum of tag associations $r(z,\omega)$ of each related product tag $z \in \mathbb{Z}_p$ with the preference tag ω, assuming that the primitive concept overlaps between product tags are minimized $\bigcap_{z \in \mathbb{Z}_p} \mathbb{Q}_z \rightarrow 0$, such that $\epsilon \rightarrow 0$.*

Proof. A product is defined as the union of associated *primitive concepts* that product tags represent. Therefore the aggregated association for $z \in \mathbb{Z}_p$ is:

$$\begin{aligned}\eta(p,\omega) &= \frac{|\mathbb{Q}_p \cap \mathbb{Q}_\omega^+|}{|\mathbb{Q}_\omega^+|} - \frac{|\mathbb{Q}_p \cap \mathbb{Q}_\omega^-|}{|\mathbb{Q}_\omega^-|} \overset{Eq.\ 6}{\Leftrightarrow} \\ \eta(p,\omega) &= \frac{|(\bigcup_z \mathbb{Q}_z) \cap \mathbb{Q}_\omega^+|}{|\mathbb{Q}_\omega^+|} - \frac{|(\bigcup_z \mathbb{Q}_z) \cap \mathbb{Q}_\omega^-|}{|\mathbb{Q}_\omega^-|} \Leftrightarrow \\ \eta(p,\omega) &= \frac{|\bigcup_z (\mathbb{Q}_z \cap \mathbb{Q}_\omega^+)|}{|\mathbb{Q}_\omega^+|} - \frac{|\bigcup_z (\mathbb{Q}_z \cap \mathbb{Q}_\omega^-)|}{|\mathbb{Q}_\omega^-|}\end{aligned} \tag{8}$$

Each nominator can be further analyzed using the general form of the Inclusion-Exclusion principle [16]. The first fraction nominator is expanded as, for any subset of product tags $\emptyset \neq \mathbb{Z}'_p \subseteq \mathbb{Z}_p$, and then all intersection between a preference tag and more than one product are isolated to determine overlaps:

$$\begin{aligned}\left|\bigcup_z (\mathbb{Q}_z \cap \mathbb{Q}_\omega^+)\right| &= \sum_{\emptyset \neq \mathbb{Z}'_p \subseteq \mathbb{Z}_p} \left[(-1)^{|\mathbb{Z}'_p|-1} \left| \bigcap_{z \in \mathbb{Z}'_p} \mathbb{Q}_z \cap \mathbb{Q}_\omega^+ \right|\right] \\ &= \sum_{z \in \mathbb{Z}_p} |\mathbb{Q}_z \cap \mathbb{Q}_\omega^+| \\ &\quad \underbrace{+ \sum_{\substack{|\mathbb{Z}'_p|>1 \\ \mathbb{Z}'_p \subseteq \mathbb{Z}_p}} \left[(-1)^{|\mathbb{Z}'_p|-1} \left| \bigcap_{z \in \mathbb{Z}'_p} \mathbb{Q}_z \cap \mathbb{Q}_\omega^+ \right|\right]}_{\epsilon^+}\end{aligned} \tag{9}$$

Thus:

$$\left|\bigcup_z (\mathbb{Q}_z \cap \mathbb{Q}_\omega^+)\right| = \sum_{z \in \mathbb{Z}_p} |\mathbb{Q}_z \cap \mathbb{Q}_\omega^+| + \epsilon^+ \tag{10}$$

Respectively it is shown that:

$$\left|\bigcup_z (\mathbb{Q}_z \cap \mathbb{Q}_\omega^-)\right| = \sum_{z \in \mathbb{Z}_p} |\mathbb{Q}_z \cap \mathbb{Q}_\omega^-| + \epsilon^- \tag{11}$$

The terms ϵ^+ and ϵ^- are the overlap correction terms introduced by the Inclusion-Exclusion principle. These terms express the semantic overlap between different product tags of a product and a single preference tag. Therefore, it is possible now to expand *Relation* 8:

$$\begin{aligned}\eta(p,\omega) &= \frac{\sum_z |\mathbb{Q}_z \cap \mathbb{Q}_\omega^+| + \epsilon^+}{|\mathbb{Q}_\omega^+|} - \frac{\sum_z |\mathbb{Q}_z \cap \mathbb{Q}_\omega^-| + \epsilon^-}{|\mathbb{Q}_\omega^-|} \overset{Eq.\ 1\ \&\ 3}{\Leftrightarrow} \\ \eta(p,\omega) &= \sum_z r^+(z,\omega) - \sum_z r^-(z,\omega) + \epsilon \overset{Eq.\ 5}{\Leftrightarrow} \\ \eta(p,\omega) &= \sum_z r(z,\omega) + \epsilon\end{aligned} \tag{12}$$

where:

$$\epsilon = \frac{\epsilon^+}{|\mathbb{Q}_\omega^+|} + \frac{\epsilon^-}{|\mathbb{Q}_\omega^-|} \tag{13}$$

Since the intersection between sets is a commutative operation, it can be derived from *Eq.* 9 that:

$$\left|\bigcap_{z \in \mathbb{Z}'_p} \mathbb{Q}_z \cap \mathbb{Q}_\omega^+\right| = \left|\mathbb{Q}_\omega^+ \cap \left(\bigcap_{z \in \mathbb{Z}'_p} \mathbb{Q}_z\right)\right| \tag{14}$$

Equations 9, 10 and 11 show that if the overlaps $\bigcap_{z \in \mathbb{Z}_p} \mathbb{Q}_z \to 0$ between product tags are minimized then $\epsilon^+, \epsilon^- \to 0 \Rightarrow \epsilon \to 0$. Thus the lemma is proved. □

For example assume the product $p=$ "orange-lettuce-rice salad" in *Supplementary Figure* S.2c, which is associated with the product tags $\mathbb{Z}_p = \{$ "vegetable", "fruit", "cereal"$\}$. The product tags z_1 = "cereal", z_2 = "fruit" and z_3 ="vegetable" share several *primitive concepts*, such as "plant part". Each product tag has a positive association score with the preference tag $\omega=$ "vegetarian". As it is showcased in *Supplementary Figure* S.2d there are several overlaps between associations, due to the shared primitive concepts of the product tags. Summing all association scores with the preference tag "vegetarian" introduces errors because of the shared "primitive concepts". The calculation of error correction terms ϵ^+, ϵ^- requires all possible combinations of intersections of product tags with shared *primitive concepts*.

Such calculation in the worst case requires an exponential time complexity of $O(2^n)$ for every aggregate association score. Therefore, the aggregation of association scores may become infeasible for an ontology with a high number of shared *primitive tags* amongst associations per product. This challenge can be addressed in the construction of the ontology, by indentifying and isolating overlapping *primitive concepts*. In the previous example, this can be achieved by creating a new product tag z_4 = "plant part" and assigning it to all products that have the "vegetable" or "fruit" tag. All the association scores between the "vegetable" and "fruit" product tags are now reduced by an amount δ, such that $r(z,\omega) = r(z,\omega) - \delta$ with $z \in \{z_1, z_2\}$. All associated preference tags with the product tags "fruit" and "vegetable" can now be associated to the product tag "plant part" with association score $r(z_4,\omega) = \delta$, as illustrated in *Supplementary Figure* S.2e. In such case, all terms of *Eq.* 13 are equal to 0, since all possible intersection are equal to the empty set.

Based on the above example to avoid overlaps, a generic **reduction design principle** is proposed during the ontology design:

1) If a product tag contains all the *primitive concepts* of another product tag, then only one is chosen and assigned to a product.
2) If there are overlaps of *primitive concepts* between two product tags of the same product, but neither can be omitted because their non-shared *primitive concepts* are important, then the intersection of their *primitive concepts* should be treated as a separate product tag and assigned to all products these tags are associated with. The shared *primitive concepts* are omitted from the original product tags.

The introduction of the reduction design principle minimizes overlap error $\epsilon \to 0$ and therefore the aggregate association can now be calculated as:

$$\eta(p,\omega) = \sum_{z} r(z,\omega) \tag{15}$$

This calculation has linear complexity $O(n)$ to the number of product tag concepts in each aggregate association calculation. Yet, the reduction design principle methodology introduces a quadratic $O(n^2)$ complexity in regards to product tags, once during the creation of the ontology, as each tag needs to be compared against each other to determine overlaps. The reduction design principle is illustrated in *Supplementary Figure* S.2e.

If the aggregated association is 0, then it is not possible to determine whether a product supports or opposes a preference tag, since it contains equally enough positive and negative concepts. The uncertainty is treated as a product having no information. Analyzing and comparing the aggregate associations between different preferences can be used for the identification of possible *trade-offs* and *rebound effects*. If no overlapping occurs ($\epsilon = 0$) between the associations, then the individual bounding property is also extended to the aggregated association. The **aggregated association bounding property** is the following:

$$\begin{gathered} \frac{|\mathbb{Q}_p \cap \mathbb{Q}_\omega^+|}{|\mathbb{Q}_\omega^+|} - \frac{|\mathbb{Q}_p \cap \mathbb{Q}_\omega^-|}{|\mathbb{Q}_\omega^-|} \in [-1,1] \overset{\textit{Eq. } 12, \epsilon=0}{\Leftrightarrow} \\ \eta(p,\omega) \in [-1,1] \end{gathered} \tag{16}$$

Application of the reduction design principle reduces overlaps, yet it may increase the amount of tags and associations in the ontology, when tags are decomposed to disjoint tags with less primitive concepts. Furthermore, successful application relies on the ability of the expert or system to identify and break down associations between preference and product tags. Therefore a trade-off is introduced between efficiency, performance and maintenance of the ontology.

SM.2 Product Rating Mechanism

The ontology is used to calculate a distributed and privacy-preserving product rating value $\varrho(p,u) \in \mathbb{R}$ between a product p and a user u. This is achieved by implementing a product rating system with rating design principles of the content based recommender systems [17], [18]. The product rating is designed to use the aggregated association scores $\eta(p,\omega)$.

1) Comparable aggregated associations

Different products may satisfy the same preference tag via product tags that are related to different *primitive concepts*. For that reason, comparison of aggregated associations for the same preference tag and different products are not easy to interpret, i.e. each product may have completely unique characteristics that satisfy each preference. To be able to compare how different products satisfy a preference, a reference product can be used to normalize all aggregate association scores. The reference products are defined as follows: (i) a reference product $p+$ that maximally satisfies the user's preferences and (ii) a reference product $p-$ that maximally opposes them. For example, a reference product can be a theoretical or existing product that contain all ontology product tags related to a preference tag. The maximum possible positive aggregated association score product is defined as the aggregated association score of a product that contains all product tags positively associated with a preference tag:

$$\begin{gathered} \eta^+(\omega) = \sum_{z} r(z,\omega), \\ \{z | z \in \mathbb{Z}_{p+} \wedge r(z,\omega) > 0\} \end{gathered} \tag{17}$$

Such product is referred to as *positive reference product*, and its association score is the *positive reference association*.

Following the same principle, a product with the minimum possible negative aggregated association score is also introduced. Such product is referred to as *negative reference product*, and its association score is the *negative reference association*. The calculation for such score is:

$$\eta^{-}(\omega)=\sum_{z} r(z,\omega), \quad \{z|z\in\mathbb{Z}_{p-}\wedge r(z,\omega)<0\} \tag{18}$$

Once the aggregated association scores are calculated, a normalization is applied by dividing with the preference scores related to a preference tag. Therefore, comparison between different products is possible:

$$\eta^{*}(p,\omega)=\begin{cases}\frac{\eta(p,\omega)}{|\eta^{-}(\omega)|}, & \eta(p,\omega)<0\\ 0, & \eta(p,\omega)=0\\ \frac{\eta(p,\omega)}{\eta^{+}(\omega)}, & \eta(p,\omega)>0\end{cases} \tag{19}$$

Existence of overlaps between product tags when calculating the aggregated association of theoretical reference scores based causes the normalized aggregated association to approach 0, as the denominator in *Eq.* 19 increases. Still, it is guaranteed that support or opposition towards a preference tag are not switched due to the overlaps in normalization, since the denominator is positive in both *Relations* 17 and 18. The normalized association score is bound in the range $[-1,1]$, since an actual product may have at most the maximum positive or minimum negative aggregated association score towards a preference tag. Therefore, the term $\eta^{*}(p,\omega)$ is also bound in range $[-1,1]$. The choice of the reference products is left to the ontology designer. Possible choices for a reference product are:

1) an existing product that achieves the highest/negative aggregate association score.
2) a theoretical or existing product that shares all positive/negative associations with a preference tag, while respecting the reduction design principle.
3) a theoretical or existing product that shares all positive/negative associations with a preference tag, without respecting the reduction design principle. This option calculates the highest possible denominator value in *Eq.* 19. This clipping mechanism is illustrated in *Supplementary Figure* S.3.

The above choices were evaluated with the app testers, and the normalized aggregated scores produced with the third choice were used during the ASSET field test.

*2) **Sustainability Index: Non-personalized Product Representations***

The next step to calculate the product ratings is to establish a relationship between preferences and products. Aggregation over the normalized association score of a product and preference tags of a preference state estimates this relationship. This aggregation is performed by using a measure of central tendency as the estimator. More specifically, here the expected value is chosen. The expected value of the normalized aggregated association scores of all the preference tags that are related to the preference is an estimate that quantifies the support or opposition of a product by the preference:

$$v(p,c)=\frac{\sum_{\omega\in\Omega_c}\eta^{*}(p,\omega)}{|\Omega_c|} \tag{20}$$

Each preference tag represent a sustainability goal considered in a preference statement. When a product has positive normalized aggregated association score with a preference tag, then it supports in achieving the sustainability goal represented by that tag. In the opposite case, the product may cause the failure of achieving a sustainability goal. Therefore, the above calculation is referred to as the sustainability index of a product for a preference, as it indicates whether purchasing a product supports in achieving or failing a sustainability goal. Calculating the expected preference-product associations for all preferences and a product yields a numerical vector representation for that product in the preference semantic space. Such representations are used to compare products and determine whether a product is expected to be supported or opposed by a set of preferences. Measures of central tendency over a product representation, such as the mean $\overline{v(p,c)}$ can be used to calculate the non-personalized ontology estimate of the product sustainability.

*3) **Self-determined User Personalization***

High preference scores indicate that a preference is important for a user when calculating the product rating. Therefore, a user's preference scores are used as weights of importance for each preference. A user can express both opposition and support towards any preference. Thus, any inaccuracy or bias introduced by the ontology design may be mitigated by the user by adjusting preference scores. This is also considered as an implicit user-determined extra correction on overlapping *primitive concepts*. Opposition and support of a user towards preferences are modeled via the offset of a preference score from the preference score median $\overline{s}$ as shown in *Eq.* 21:

$$o(s_{u,c})=s_{u,c}-\overline{s} \tag{21}$$

The higher the support or opposition of a user towards a preference, the higher the absolute value of the offset.

A weighted average between product-preference association scores and the user preference offsets are used to calculate a personalized association between a user and a product, given the user's self-determined preference scores. The sum of absolute offsets is used as denominator to preserve the sign of the rating while normalizing. Preserving the signs allows to extend the association logic to user-product level. Positive product ratings indicate that the product mostly supports the user supported preferences and opposes user opposed

preferences. For negative product ratings, the product opposes user supported preferences and supports user opposed preferences. Although self-determined personalization allows for a user's *subjectivity* to influence the ratings, the user is made aware about which preferences produce such ratings. This results in a learning effect, that increases user awareness towards their sustainability preferences. For example when a person tunes the preferences to allow their favorite products achieve high scores, they are aware that to do so, they have to go against the preference they originally support. Thus the unscaled product rating is calculated as follows:

$$\varrho^*(p,u) = \frac{\sum_c v(p,c) \cdot o(s_{u,c})}{\sum_c |o(s_{u,c})|} \tag{22}$$

The scale of $[-1,1]$ can be transformed to any range of real numbers by applying a linear scaling with parameters α and β. Transforming the rating scale has several applicability scenarios. The rating can be scaled to different ranges to match the most preferred grading system of the country, where the algorithm is deployed [19]. Another possible usage of the scaling coefficients is to attribute rating for asymmetric perception of negative and positive rating values [20], [21]. For example, based on the work of Parguel et al. [21], negative values have a higher impact on user perception. In such case, a different lower scaling coefficients α,β can be used to reduce the impact.

$$\varrho(p,u) = \alpha \cdot \rho^*(p,u) + \beta \tag{23}$$

The product rating scale is designed to utilize user preference scores and create an association between a user and a product based on the product-preference associations. The rating value is expected to be bounded in a range $[\beta-\alpha,\beta+\alpha]$. Products that neither support nor oppose the user's preferences are assigned the mean value of the range β. If the product supports a user supported preference or opposes a user opposed preference, then the product rating increases. On alternative scenarios the product rating decreases.

The rating value compresses *overwhelming information* and shows to the user an estimate of the personalized product sustainability. Depending on the UI design, it is possible to allow the user to further explore the ontology dynamics that result in this rating value. In the *Supplementary Figure* S.1 all related ontology entities and rating calculations are presented.

*4) **Algorithm complexity***

All calculations to compute the product-preference representations rely on information that is not related to the user. Therefore, *Equations* 15-20 can be computed without privacy intrusion risks. The computational cost is significantly reduced for the user's device, since it is possible to calculate, store and distribute the product representations $v(p,c)$ by using a central database system. The calculation and storage complexity for the worst case scenario, where all products are connected to every product tag, all product tags are associated to all preference tags and all preference tags are connected to all preferences is:

$$O(n) = P \cdot T \cdot \Omega \cdot C \tag{24}$$

where:

P : The number of all products.

T : The number of all product tags.

Ω : The number of all preference tags.

C : The number of all preferences.

Assuming that all the above sizes are equal, the worst case computational and storage complexity for the algorithm is polynomial to the power of 4:

$$(\text{Relation } 24) \wedge (P = T = \Omega = C) = O(n^4) \tag{25}$$

Since the total products and product tags are often significantly more than the preference statements and preference tags, the expected time and space complexity is reduced to quadratic polynomial time $O(n^2)$. Therefore, modern CPUs on mobile phones and database servers can handle up to hundreds of thousands of product rating calculations per minute and store several thousands of products and product tags [22].

*5) **Overlaps and possible errors***

As discussed in *Supplementary Section* SM.1, the application of the reduction design principle introduces several trade-offs between the efficiency of the rating calculations and maintenance of the ontology. Time constraints and limited resources regarding the construction and testing of the ontology may allow overlaps in the ontology, which introduce errors and biases that may break the bounding properties of *Equation* 5. Thus, in such cases a clipping normalization is introduced to avoid numerical instabilities due to overflows outside the theoretical aggregated association bounds:

$$g(a) = \begin{cases} -\tau & ,\text{if} \quad a \leq -\tau \\ a & ,\text{if} \quad -\tau < a < \tau \\ \tau & ,\text{if} \quad a \geq \tau \end{cases} \tag{26}$$

The introduction of the clipping changes *Relations* 15, 17 and 18 to the following:

$$\eta(p,\omega) = g\Big(\sum_{z \in \mathbb{Z}_p} r(z,\omega)\Big) \tag{27}$$

$$\eta^{+}(\omega)=g(\sum_{z} r(z,\omega)),\quad \{z|z\in\mathbb{Z}\wedge r(z,\omega)>0\} \tag{28}$$

$$\eta^{-}(\omega)=g(\sum_{z} r(z,\omega)),\quad \{z|z\in\mathbb{Z}\wedge r(z,\omega)<0\} \tag{29}$$

Even if overlaps affect the rating process, the users are able to mitigate the error by readjusting their preference scores, introducing an extra correction mechanism from their side. An illustration of the clipping mechanism on the normalized aggregate score is found in *Supplementary Figure* S.3 in step 1.

SM.3 **Tractable And Explainable Ratings**

The proposed ontology design and rating calculations rely on a fully tractable analytical framework. It is possible to calculate the exact amount that a preference, preference or product tag contributed to the rating. More specifically following eqs. (22) and (23), one could solve to calculate the exact contribution of a specific preference c^* to the product rating as follows:

$$\varrho(p,u)=\alpha\left(\frac{\sum_{c\in\mathbb{C}-\{c^*\}} v(p,c)\cdot o(s_{u,c})}{\sum_c |o(s_{u,c})|} + \frac{v(p,c^*)\cdot o(s^*_{u,c})}{\sum_c |o(s_{u,c})|}\right)+\beta \tag{30}$$

Therefore the contribution of preference c^* to the final rating is calculated as:

$$\alpha\frac{v(p,c^*)\cdot o(s^*_{u,c})}{\sum_c |o(s_{u,c})|} \tag{31}$$

The contribution of a specific preference tag ω^* is calculated following the same decomposition logic on eqs. (20), (22) and (23), the contribution of a specific preference tag to the rating can be calculated as:

$$\alpha\frac{\sum_{c\in\mathbb{C}}\frac{\eta^*(p,\omega^*)}{|\Omega_c|}\cdot o(s_{u,c})}{\sum_c |o(s_{u,c})|} \tag{32}$$

And the contribution of a specific product tag z^* to the product rating can be calculated by decomposing eqs. (19), (20), (22) and (23):

$$\alpha\frac{\sum_{c\in\mathbb{C}}\frac{\sum_{\omega\in\Omega_c}\frac{r(z^*,\omega)}{\eta_{norm}}}{|\Omega_c|}\cdot o(s_{u,c})}{\sum_c |o(s_{u,c})|} \tag{33}$$

where η_{norm} has the corresponding denominator value of either $\{\eta^{+}(\omega),\eta^{-}(\omega)\}$ that normalizes the aggregated association in eq. (19). In case that clipping is used, as shown in *Eq.* 27, the contribution is calculated proportionally:

$$\alpha\frac{\sum_{c\in\mathbb{C}}\frac{\sum_{\omega\in\Omega_c}\frac{\frac{r(z^*,\omega)}{\sum_z r(z,\omega)}}{\eta_{norm}}}{|\Omega_c|}\cdot o(s_{u,c})}{\sum_c |o(s_{u,c})|} \tag{34}$$

Product tag contributions can also be calculated per preference or preference tag. Such calculations are possible by ommitting the calculation related to the product rating and calculate the amount a product tag contributes to other scores, such as the sustainability index discussed in Equation (20). Finally, contributions can either be stored when calculating the product rating, increasing memory complexity or recalculated after the rating calculation, thus increasing computation complexity.

SM.4 **Crowd Sourced Product Data**

To facilitate wisdom of the crowds, a datathon took place at the University of Tartu, Estonia on November 2017 [23]. In total three teams of 3-4 people participated in the datathon, and produced data files and code within 72 hours. The datathon teams processed text data from Wikipedia pages related to product characteristics and sustainability goals. Each team used statistical methods and machine learning techniques based on NLP and sentiment analysis to extract an association score between product tags and sustainability topics. Each score should cite the wikipedia page id that was used to extract such data. This resulted in several thousand association scores between product and preference tags. The ASSET consortium used the majority of these results to evaluate existing association score and discover new ones.

Several new product tags and associations were introduced in the ontology, mainly in the environmental and social categories. Further collaboration with members of the winning datathon team took place in order to enrich and evaluate the created ontology.

As new product tags and association scores were discovered, the datathon provides a real world example on how machine learning can be used to populate a sustainability ontology. Furthermore, the datathon showcases a working case, where crowd-sourced data can be used for populating a sustainability ontology.

SM.5 **Software Libraries**

Data were collected, stored and processed for categorization and classification by AINIA according to GDPR. The smart phone app integrates a library version [24] of the Nervousnet system [25]. While no Nervousnet functionality has been used during the lifetime of this project and work, its integration has been part of a deliverable for the ASSET EU-project. Its modular and flexible sensor data management system has potential future applicability in shopping scenarios from multiple retailer shops.

For the development and analysis of the presented results several libraries were used. Some notable references are: (i) pycausalimpact[2], (ii) scikit-learn [27], (iii) scipy [28], (iv) the discrete information theory package [29], (v)pyclustering [30], (vi) plotly [31]. Several other libraries that were used, are also mentioned in the paper code repository.

SUPPLEMENTARY NOTES

SN.1 **Entry And Exit Survey**

Entry and exit surveys are presented to the user during the field test. The aim of the surveys is to collect data regarding user opinions on sustainable consumption, sustainability preferences and the usage of the application. The number of participants for each survey is summarized in *Supplementary Table* S.8 Answers on the exit survey provide useful insights regarding the behavioral change and sustainability awareness of users during the field test. Users confirmed that the rating methodology offers product ratings that justify their preference settings. Access to product information and the product tags is visible in the application for users that click on a product. Such information assists the decision or the product purchase. User declared that it is relatively easy to access the product information and that the provided information justifies the rating (*Supplementary Figures* S.5a and S.5b). The highly rated products tend to match the preferences of the majority of the users as shown in *Supplementary Figures* S.5c, S.5e and S.5f. Finally, an increase in discovery of novel products is stated by the users in *Supplementary Figure* S.5d.

Shopping behavior is affected for some users by using the app. Mainly in retailer B users support that they would not buy products that achieve low ratings and buy high rated products before using the app, which emphasizes their sustainability focus (*Supplementary Figures* S.6a and S.6a). Regarding future purchases, users support that they will buy products that achieve high ratings and also avoid buying products that achieve low rating, as shown in *Supplementary Figures* S.6c and S.6d. Furthermore, users in retailer A estimated that they buy over 5% of highly rated products in retailer A, whereas most retailer B users estimate a value over 10%, as illustrated in *Supplementary Figure* S.8. In both retailers, the majority of users agree to an extent that the usage of the application raised their awareness towards sustainability *Supplementary Figure* S.6e

Product information and price seem to be the main decision factors when users decide regarding a product purchase, as illustrated in *Supplementary Figure* S.7.

Concluding, weakly qualitative evaluation indicates that the application provides enough information to the users to purchase highly rated products, thus increasing the sustainability of their consumption. Screenshots from the app UI of the survey and preferences is illustrated in *Supplementary Figure* S.11. Users perceive that the product rating takes into account their preferences and is well justified by the available data. Other factors may affect the participation of users such as technical problems with the app, low usability of UI, seasonality (traveling), limited access to retailers shops for some consumers, etc.

SN.2 **Evaluation Of Product Prices**

Sustainability index vs product price

This evaluation studies whether products that highly support a specific preference have a higher price compared to products from the same category. Evaluating sustainability index and price over all products provides insights about the general expenditure. Consumers might be interested in replacing products they buy with more sustainable products from the same category. To evaluate whether more expensive products achieve higher sustainability scores compared to competitive products from the same category, both price per unit and sustainability index are rescaled using the min-max normalization for each product per category. I.e. Each value is normalized by substracting the minimum value and then dividing by the difference between maximum and minimum value [26]. For the normalization to work, each category needs to contain at least two products with different sustainability index and price values. Therefore, only such categories are used for the analysis. As illustrated in *Supplementary Figure* S.9a, purchasing more expensive products per category in retailer A seems to have almost no impact on the sustainability index for most categories. The vegan diet preference "H.12" is negative correlated with price, possibly indicating that the most expensive products per category often contain an animal product. For retailer B, the sustainability index for most preferences is either positively correlated or uncorrelated with prices. Negative correlated preferences

[2]https://github.com/dafiti/causalimpact

with prices are "E.2", "E.3" and "S.1", indicating that one can purchase cheaper products per category, which are farmed in a sustainable manner, distributed in an environmentaly friendly way and passed sustainability auditing process. This result may indicate that in most categories the most expensive product originates from a place that the transportation has high environmental impact, sustainable farming techniques are not followed or sustainable auditing processes do not hold. Furthermore, since retailer B is a sustainability focused retailer, it is expected that the suppliers may mitigate sustainable production costs in their product prices.

Note that the lower expenditure level of Retailer A during the summer is a seasonality effect that is prominent due to the students' holidays and constructions in the neighborhood.

References

[1] National Research Council et al. Recommended dietary allowances*. *Nutrition Reviews* **1**, 164–168 (1943).
[2] Compare sustainability of brands | buy sustainable | Rank a Brand. https://rankabrand.org/ (2020). Accessed in April 2020.
[3] GoodGuide. https://www.goodguide.com/ (2020). Accessed in April 2020.
[4] Shop Ethical! | Your ethical consumer guide. https://www.ethical.org.au/3.4.2/ (2020). Accessed in April 2020.
[5] Mobile Phone Ethical Comparison. https://thegoodshoppingguide.com/ethical-mobile-phones (2020). Accessed in April 2020.
[6] The Green Stars Project | User-generated ratings for ethical consumerism. https://greenstarsproject.org/ (2020). Accessed in April 2020.
[7] Home - WikiRate. https://wikirate.org/ (2020). Accessed in April 2020.
[8] Behind the brands | Change the way the food companies that make your favorite brands do business. (2020). URL https://www.behindthebrands.org/. Accessed in April 2020.
[9] Helbing, D. *Quantitative sociodynamics: stochastic methods and models of social interaction processes* (Springer Science & Business Media, 2010).
[10] Osgood, C. E., Suci, G. J. & Tannenbaum, P. H. *The measurement of meaning*. 47 (University of Illinois press, 1957).
[11] Goswami, P. & O'Haire, T. Developments in the use of green (biodegradable), recycled and biopolymer materials in technical nonwovens. In *Advances in Technical Nonwovens*, 97–114 (Elsevier, 2016).
[12] Villalba, G., Segarra, M., Fernandez, A., Chimenos, J. & Espiell, F. A proposal for quantifying the recyclability of materials. *Resources, Conservation and Recycling* **37**, 39–53 (2002).
[13] Rodriguez, M. A. & Egenhofer, M. J. Determining semantic similarity among entity classes from different ontologies. *IEEE Transactions on Knowledge and Data Engineering* **15**, 442–456 (2003).
[14] Kretchmer, N. Lactose and lactase (1972).
[15] Miller, G. & Miller, N. Plasma-high-density-lipoprotein concentration and development of ischaemic heart-disease. *The Lancet* **305**, 16 – 19 (1975). Originally published as Volume 1, Issue 7897.
[16] Rota, G.-C. On the foundations of combinatorial theory i. theory of möbius functions. *Probability theory and related fields* **2**, 340–368 (1964).
[17] Jannach, D., Zanker, M., Felfernig, A. & Friedrich, G. *Recommender systems: an introduction* (Cambridge University Press, 2010).
[18] Isinkaye, F. O., Folajimi, Y. O. & Ojokoh, B. A. Recommendation systems: Principles, methods and evaluation. *Egyptian Informatics Journal* **16**, 261–273 (2015).
[19] Blanton, H., Buunk, B. P., Gibbons, F. X. & Kuyper, H. When better-than-others compare upward: Choice of comparison and comparative evaluation as independent predictors of academic performance. *Journal of personality and social psychology* **76**, 420 (1999).
[20] Kwon, W.-S., Englis, B. & Mann, M. Are third-party green–brown ratings believed?: The role of prior brand loyalty and environmental concern. *Journal of Business Research* **69**, 815–822 (2016).
[21] Parguel, B., Benoît-Moreau, F. & Larceneux, F. How sustainability ratings might deter 'greenwashing': A closer look at ethical corporate communication. *Journal of business ethics* **102**, 15 (2011).
[22] Papadimitriou, G., Chatzidimitriou, A. & Gizopoulos, D. Adaptive voltage/frequency scaling and core allocation for balanced energy and performance on multicore cpus. In *2019 IEEE International Symposium on High Performance Computer Architecture (HPCA)*, 133–146 (IEEE, 2019).
[23] Social impact data hack 2017. https://sidh2017.ut.ee/. [Online; April 2020].
[24] nervousnet library. https://github.com/prasad79/AssetDemoUsingAAR. [Online; April 2020].
[25] Pournaras, E., Moise, I. & Helbing, D. Privacy-preserving ubiquitous social mining via modular and compositional virtual sensors. In *In 2015IEEE 29th International Conference on Advanced Information Networking and Applications*, 332–338 (IEEE, 2015).
[26] Pedregosa, F. *et al.* Scikit-learn: Machine Learning in Python . *Journal of Machine Learning Research* **12**, 2825–2830 (2011).
[27] Jones, E., Oliphant, T., Peterson, P. *et al.* SciPy: Open source scientific tools for Python (2001–). URL http://www.scipy.org/. [Online; accessed in April 2020].
[28] James, R. G., Ellison, C. J. & Crutchfield, J. P. dit: a Python package for discrete information theory. *The Journal of Open Source Software* **3**, 738 (2018).
[29] Novikov, A. annoviko/pyclustering: pyclustering 0.8.2 release (2018). URL https://doi.org/10.5281/zenodo.1491324.
[30] Inc., P. T. Plotly, plotting library. https://plot.ly (2015).
[31] Dodge, Y. & Commenges, D. *The Oxford dictionary of statistical terms* (Oxford University Press on Demand, 2006).
